# Role of neuronavigation in the surgical management of brainstem gliomas

**DOI:** 10.3389/fonc.2023.1159230

**Published:** 2023-05-02

**Authors:** Mingxin Zhang, Xiong Xiao, Guocan Gu, Peng Zhang, Wenhao Wu, Yu Wang, Changcun Pan, Liang Wang, Huan Li, Zhen Wu, Junting Zhang, Liwei Zhang

**Affiliations:** ^1^ Department of Neurosurgery, Beijing Tiantan Hospital, Capital Medical University, Beijing, China; ^2^ China National Clinical Research Center for Neurological Diseases (NCRC-ND), Beijing, China; ^3^ Beijing Neurosurgical Institute, Beijing Tiantan Hospital, Capital Medical University, Beijing, China

**Keywords:** brainstem glioma, neuronavigation, surgery, control study, single-center

## Abstract

**Objective:**

NeuroNavigation (NN) is a widely used intraoperative imaging guidance technique in neurosurgical operations; however, its value in brainstem glioma (BSG) surgery is inadequately reported and lacks objective proof. This study aims to investigate the applicational value of NN in BSG surgery.

**Method:**

A retrospective analysis was performed on 155 patients with brainstem gliomas who received craniotomy from May 2019 to January 2022 at Beijing Tiantan Hospital. Eighty-four (54.2%) patients received surgery with NN. Preoperative and postoperative cranial nerve dysfunctions, muscle strength, and Karnofsky (KPS) were evaluated. Patients’ radiological features, tumor volume, and extent of resection (EOR) were obtained from conventional MRI data. Patients’ follow-up data were also collected. Comparative analyses on these variables were made between the NN group and the non-NN group.

**Result:**

The usage of NN is independently related to a higher EOR in diffuse intrinsic pontine glioma (DIPG) (p=0.005) and non-DIPG group (p<0.001). It was observed that fewer patients in the NN group suffered from deterioration of KPS (p=0.032) and cranial nerve function (p=0.017) in non-DIPG group, and deterioration of muscle strength (p=0.040) and cranial nerve function (p=0.038) in DIPG group. Moreover, the usage of NN is an independent protective factor for the deterioration of KPS (p=0.04) and cranial nerve function (p=0.026) in non-DIPG patients and the deterioration of muscle strength (p=0.009) in DIPG patients. Furthermore, higher EOR subgroups were found to be independently related to better prognoses in DIPG patients (p=0.008).

**Conclusion:**

NN has significant value in BSG surgery. With the assistance of NN, BSG surgery achieved higher EOR without deteriorating patients’ functions. In addition, DIPG patients may benefit from the appropriate increase of EOR.

## Introduction

Brainstem gliomas are a set of gliomas originating from the midbrain, pontine, and medulla, which account for 10%–20% and 2%–4% of all intracranial tumors in children and adults, respectively (1–4). The prognosis of BSGs is unfavorable, especially for DIPG, a subtype originating mainly from the pons with a median OS of 9-12 months (5–9). In recent decades, a concerted effort has been made to improve the prognosis of BSGs. With the efforts of neurosurgeons, surgery is playing an increasingly important role in BSG treatment (10–15). Surgery aims to achieve maximal safe resection for low-grade brainstem gliomas, while cytoreduction or craniotomy biopsy for high-grade gliomas, especially for DIPG (13, 16–18). However, due to the small volume of the brainstem and the dense arrangement of structures with important neurofunctions, surgery on BSGs is associated with significant risks of postoperative morbidity and impairment of life quality. Thus, it is essential to balance the EOR and the protection of neurological function during BSG surgery (19, 20). An accurate spatial location and tumor boundary guidance is the basis of achieving this balance.

As an important intraoperative technique, NN can provide accurate spatial location guidance in real-time during the surgery, which could contribute to the balance mentioned above. Therefore, NN can be a practical tool in brainstem surgery. Nevertheless, NN is criticized for its shortcomings, such as brain shift, which makes the actual location and shape of intracranial structures mismatch with their preoperative images and directly affects navigation accuracy(21–23). Due to this shortcoming, some neurosurgeons prefer to focus their attention on intraoperative neurophysiological monitoring. Hence, the application of NN is narrowed, and its applicational value in brainstem surgery is still absent of enough objective supporting evidence.

Aiming to clarify the application value of NN in BSG, we carried out this comparative analysis of outcomes of patients who underwent surgeries with and without NN, including the EOR, KPS, cranial nerve functions, muscle strength, and survival conditions.

## Materials and methods

### Study design and patient inclusion

The study was approved by the Institutional Review Board (IRB) of Beijing Tiantan Hospital, Capital Medical University. This study retrospectively screened patients with brainstem gliomas who received surgery at Beijing Tiantan Hospital from May 2019 to January 2022. The inclusion criteria were: 1) originating from the brainstem; 2) received craniotomy surgery; 3) pathologically confirmed glioma. The exclusion criteria were: 1) patients with previous treatment, including craniotomy, radiotherapy, and chemotherapy; 2) patients with unavailable medical and radiological data. Because of the nature of the retrospective study, informed consent was waived by the IRB.

### Clinical data

Data related to preoperative baseline and surgical outcomes were collected. The preoperative baseline data comprises demographic information, duration of symptoms, and preoperative manifestations. The surgical outcomes data includes postoperative short-term manifestations and prognosis. The clinical manifestation includes KPS, cranial nerve dysfunction, and muscle strength. Follow-ups were performed routinely by telephone calls or outpatient visits at least once every three months, and the follow-up results were stored in the database. All data on prognosis were obtained from this database. The deterioration of postoperative manifestation was defined as a worse condition at discharge than the preoperative time point. If patients were in the hospital for more than 14 days, the deterioration of postoperative manifestation was defined as a worse condition on the 14th day after surgery than the preoperative time point.

### Radiological assessment

All MRI scans were obtained using a 3.0T system (Magnetom TIM Trio 3.0, Siemens, Erlangen, Germany) and included T1-weighted imaging (T1WI), T2WI, fluid-attenuated inversion recovery (FLAIR) imaging, contrast-enhanced T1WI (CE T1WI), and diffusion tensor image (DTI). The radiological features, including location and imaging characteristics, were obtained from the medical imaging data. Based on radiological features, the BSGs were classified into four types according to the Choux classification (24). The definition of DIPG is that the tumor arises from the pons, exhibits a diffuse pattern of involvement, hypointensity in T1WI, hyperintensity in T2WI and Flair, and involves ≥50% of the pons (5). With the assistance of 3D Slicer software, preoperative and postoperative tumor volumes were quantified on T1WI (25). Two experienced neurosurgeons blinded to the patient information performed the assessments above. If the conclusion differed substantially, they would reappraise the patient information and communicate mutually until a consensus was reached. The EOR was then calculated using the equation: 100 − (postoperative volume/preoperative volume × 100) with the result expressed in the percentage of resection.

### Surgical indications and protocol

For BSGs, the surgery strategy aims to achieve the maximal extent of safe resection. Nevertheless, gross total resection is unfeasible and the benefit of surgery still needs further investigation in DIPG. In this study cohort, the surgical strategy for DIPGs is to perform a craniotomy biopsy or cytoreductive surgery within a functional safe area, especially for the contrast-enhanced area or PET hypermetabolic area. Two illustrated cases were included in the (**Supplementary material Figure S1** and **S2**).

With comprehensive consideration of preoperative clinical manifestation and MRI findings, the surgical approach and corresponding safe entry zone were determined individually. The preoperative MRI findings of the patient include the location, size, growth pattern, and pattern of contrast enhancement of the tumor, and the location of the vital fiber bundles (such as bilateral corticospinal tracts and medial lemniscus) indicated by diffusion tensor tractography.

**Figure 1:**
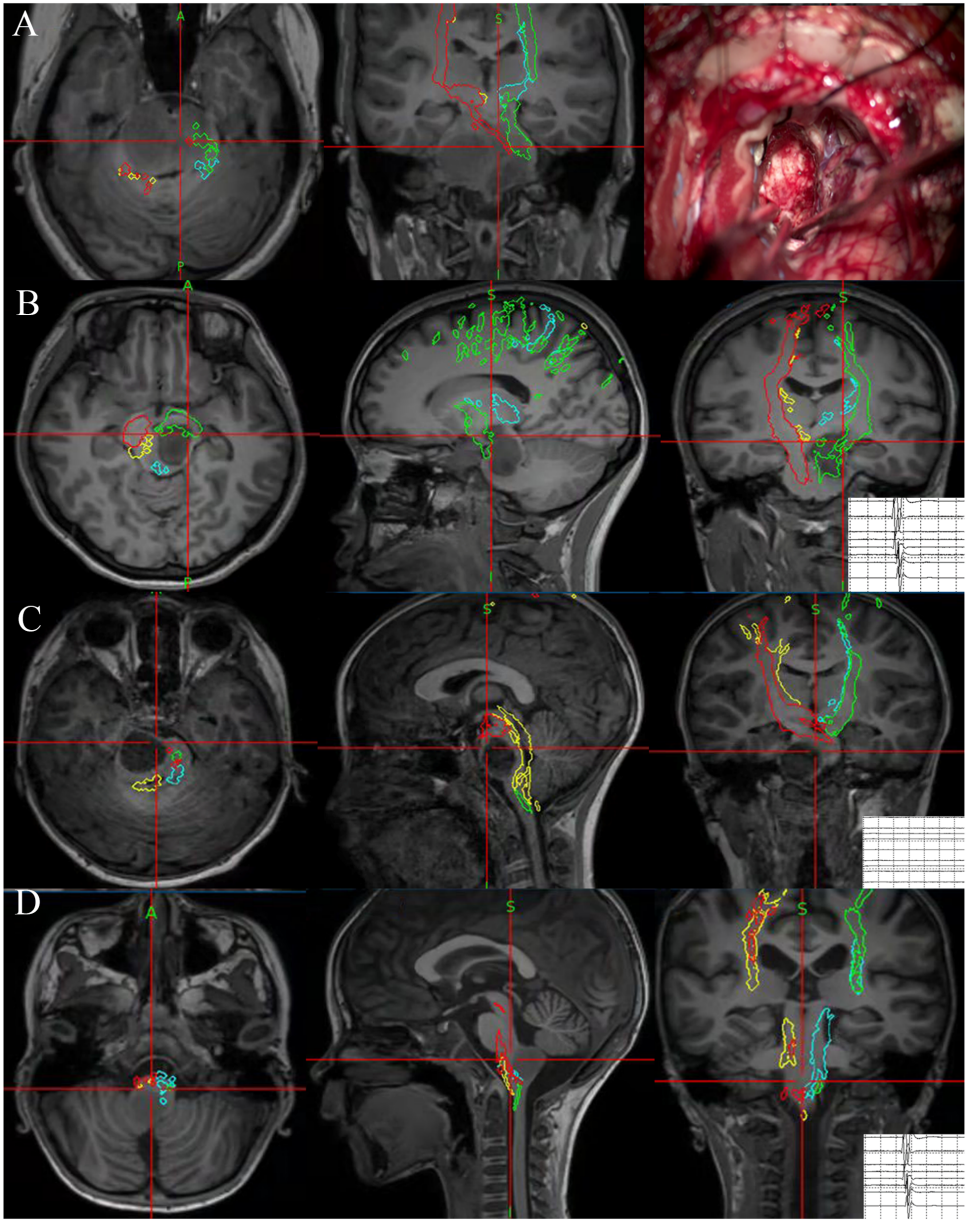
Illustration of four cases that underwent NN-guided surgery for BSG. NN indicated the cytoreductive surgery in DIPG. The predetermined surgical boundaries located near the CST were reached under navigation guidance. During operation, neurophysiological monitoring lost its value because the CST was severely disrupted preoperatively **(A)**. A focal glioma located in midbrains. NN indicated the current position was near CST and thus EOR was reached, and neurophysiological monitoring also reported CST located near the current position **(B)**. A focal glioma located in pontine. NN indicated the position was near the imaging boundary of a tumor in the pons, and neurophysiological monitoring could not indicate the nearest CST as there is a distance between current position and the nearest CST **(C)**. A focal glioma located in medulla oblongata. NN indicated the position was near the imaging boundary of a tumor in the medulla, neurophysiological monitoring also reported CST located near the current position **(D)**.

Every surgery was performed under the premise of using standard microsurgical techniques and electrophysiological monitoring. Whether or not the surgeries were performed with NN is determined by the neurosurgeon based on the comprehensive consideration of surgical strategies. For surgeries with NN, a 3D T1-weighted anatomical sequence was collected on the day of surgery and the data was imported into StealthStation S7 optical surgical navigation system (Medtronic) as a reference sequence. Tumor’s contour and tractography results were co-registered with the reference images. Repeated guidances with NN were performed from the opening of the dura mater to the completion of resection, especially during the procedure of tumor exposure and excision, to assist in getting closer to the lesion and determining the EOR and tumor boundary. During the operation, the accuracy of bony landmarks and anatomical parts of the brain stem was checked periodically. A few cases operated under the guidance of NN are illustrated in **Figure 1**.

The boundary of the surgical resection during the operation is determined by the combination of the judgment of neurosurgeons, the change of amplitude of neurophysiological monitoring, and the real-time position indicated by NN. If the neurosurgeon believes that a prespecified resection boundary has been reached, or neurophysiological monitoring indicates a risk of neurological impairment, NN indicates an unresectable site, or a prespecified surgical boundary, the surgeon would stop his manipulation.

### Statistical analysis

All statistical analyses were performed using SPSS Statistics 26.0 (IBM, Armonk, New York, USA), GraphPad PRISM 8.3.0. (GraphPad Software Inc, San Diego, CA, USA), and R (version 4.1.14). For measurement data, if the variables follow a normal distribution, means and standard deviations were calculated; t-tests or ANOVAs were used for intergroup comparisons. Otherwise, quartiles were calculated, and the Mann-Whitney U-test or Kruskal-Wallis H-test was used for intergroup comparisons. Frequencies and percentages were calculated for categorical data, and chi-square tests were used for intergroup comparisons. The Chi-square test was used to analyze the differences in the percentage of deterioration of symptoms between groups. Univariate and Multivariate linear and logistic regression were used to analyze the effect of NN on EOR and the deterioration of symptoms, respectively. Kaplan-Meier was used to compare the difference in survival between groups. Univariate and Multivariate Cox regressions were used to determine the relationship between the assistance of NN and survival. The statistically significant was set as a p < 0.05.

## Results

### Summary of included patients

A total of 170 patients met the inclusion criteria, but 15 patients fit the exclusion criteria. Finally, 155 patients were included, among which 84 underwent BSG surgery with the assistance of NN.

As DIPG and non-DIPG tumors harbor different biological and clinical features, the strategy of surgery can differ. The analyses were performed in DIPGs and non-DIPGs, respectively. Preoperative baseline variables were observed distributed balanced between NN and non-NN subgroup in the non-DIPG patients, and only preoperative symptoms were observed worse in the NN subgroup in DIPG patients (**Tables 1**, **2**).

**Table 1:** The summary of included patients (non-DIPG).

	Navigation (N=37)	Non-navigation (N=53)	P
Age (years old, median [IQR])	29 (12.5,37.5)	32 (17.5,43.5)	0.215
Gender (Female/Male)	18/19	31/22	0.356
Duration of symptoms (months, median [IQR])	5 (2.75,12)	4.0 (1,12)	0.180
Preoperative KPS (median [IQR])	80 (70,80)	80 (70,80)	0.472
Preoperative muscle strength (median [IQR])	5 (4,5)	5 (4,5)	0.720
Preoperative cranial nerve symptoms (N [percentage])	18(48.6%)	24(45.3%)	0.753
Preoperative hydrocephalus (N [percentage])	9(24.3%)	18(33.9%)	0.326
Location (N [percentage])			0.426
Midbrain	14 (37.8%)	18 (33.9%)	
Pons	8 (21.6%)	7 (13.2%)	
Medulla	15 (40.6%)	28 (52.9%)	
Preoperative tumor volume (mm^3^, median [IQR])	8586.37 (5604.39,17259)	7000.16 (3855.03,11806.85)	0.100
Choux classification (N [percentage])			0.329
Diffuse	16 (43.2%)	25 (47.2%)	
Focal	12 (32.4%)	11 (20.8%)	
Exophytic	8 (21.6%)	17 (32.0%)	
Cervicomedullary	1 (2.8%)	0	
EOR ([percentage], [IQR])	80.5% (68.9, 88.1)	58.8% (46.7,71.9)	<0.001
Postoperative tumor volume (mm^3^, median [IQR])	1323.41 (995.63,2577.38)	2301.47 (1143.51,5045.94)	0.047
Postoperative KPS (median [IQR])	70 (70,80)	70 (60,80)	0.414
Postoperative muscle strength (median [IQR])	5 (4,5)	5 (4,5)	0.414
Deterioration of cranial nerve symptom (N [percentage])	15 (40.5%)	35 (66.0%)	0.017
WHO grade (N [percentage])			0.472
I	8 (21.6%)	19 (35.8%)	
II	13 (35.1%)	16 (30.2%)	
III	1 (2.7%)	2 (3.8%)	
IV	15 (40.5%)	16 (30.2%)	
H3K27M (N [percentage])	13 (35.1%)	14 (26.4%)	0.374
Overall survival (months, median [IQR])			

**Table 2:** The summary of included patients (DIPG).

	Navigation (N=47)	Non-navigation (N=18)	P
Age (years old, median [IQR])	10 (7,19)	13 (8.75,33.25)	0.064
Gender (Female/Male)	27/20	10/8	0.890
Duration of symptoms (months, median [IQR])	2 (1,3)	2 (1,6)	0.897
Preoperative KPS (median [IQR])	70 (50,70)	70 (60,80)	0.160
Preoperative muscle strength (median [IQR])	4 (4,5)	5 (4,5)	0.014
Preoperative cranial nerve symptoms (N [percentage])	42 (89.4%)	12 (66.7%)	0.029
Preoperative hydrocephalus (N [percentage])	6 (12.8%)	3 (16.7%)	0.699
Preoperative tumor volume (mm^3^, median [IQR])	20196 (16813.5,28387.5)	15660.8 (8897.57,26561.13)	0.173
EOR ([percentage], [IQR])	57.6% (45.8, 74.5)	40.9% (32.5, 51.8)	0.003
Postoperative tumor volume (mm^3^, median [IQR])	8392.60 (5056.98,12907.2)	6011.04 (5118.49,15736.58)	0.895
Postoperative KPS (median [IQR])	50 (40,70)	60 (40,70)	0.447
Postoperative muscle strength (median [IQR])	3 (3,4)	4 (3,4)	0.272
Deterioration of cranial nerve symptom (N [percentage])	11 (23.4%)	9 (50%)	0.038
WHO grade (N [percentage])			0.357
I	1 (2.1%)	0	
II	2 (4.3%)	2 (11.1%)	
III	2 (4.3%)	2 (11.1%)	
IV	42 (89.4%)	14 (77.8%)	
H3K27M (N [percentage])	41 (87.2%)	12 (66.7%)	0.056
Overall survival (months, median [IQR])	20.5	11.5	0.042

### The assistance of NN increased EOR

Since the preoperative volume of the tumors is similar, but the postoperative volume is lower (p=0.047) in the NN subgroup in non-DIPG patients, the relationship between the assistance of NN and EOR is investigated. EOR in the NN group is significantly higher in non-DIPG patients (P<0.001, **Table 1**) as well as DIPG patients (P=0.003 **Table 2**). By performing univariate and multivariate linear regressions, it was found that NN is independently positively associated with EOR in the DIPG and non-DIPG groups (**Tables 3**, **4**).

**Table 3:** Univariate and multivariate leaner regression showing the usage of NN is an independent factor for the extent of resection in the DIPG patients.

	Univariate		Multivariate	
	t	p	t	p
Age	0.209	0.835	0.699	0.487
Gender	-0.373	0.711	-0.413	0.681
H3K27M mutation	1.084	0.282		
Navigation	2.881	0.005	2.935	0.005
WHO grade*	0.281	0.780		
Hydrocephalus	-0.328	0.744		
Preoperative cranial nerve dysfunction	-0.494	0.623		
Preoperative muscle strength	-1.008	0.317		
Preoperative KPS	-0.854	0.396		

*We regard the WHO I and II grade as control groups in WHO grade.

**Table 4:** Univariate and multivariate leaner regression showing the usage of NN is an independent factor for the extent of resection in the non-DIPG patients.

	Univariate		Multivariate	
	t	p	t	p
Age	-1.803	0.075	-1.533	0.129
Gender	0.374	0.710	1.153	0.252
H3K27M mutation	-0.715	0.477		
Navigation	5.060	<0.001	4.946	<0.001
WHO grade*	-0.800	0.426		
Location*	0.145	0.885		
Hydrocephalus	-0.519	0.605		
Preoperative cranial nerve dysfunction	-0.356	0.723		
Preoperative muscle strength	1.524	0.131		
Choux classification*	0.937	0.351		
Preoperative KPS	0.888	0.377		

*We regard the WHO I and II grade, midbrain, and diffuse type as control groups in WHO grade, Location, and Choux classification, respectively.

### The assistance of NN protects patients’ postoperative short-term quality of life

As the usage of NN increased EOR, it is vital to find out whether the increment caused more surgical-related neurological dysfunctions.

**Figure 2:**
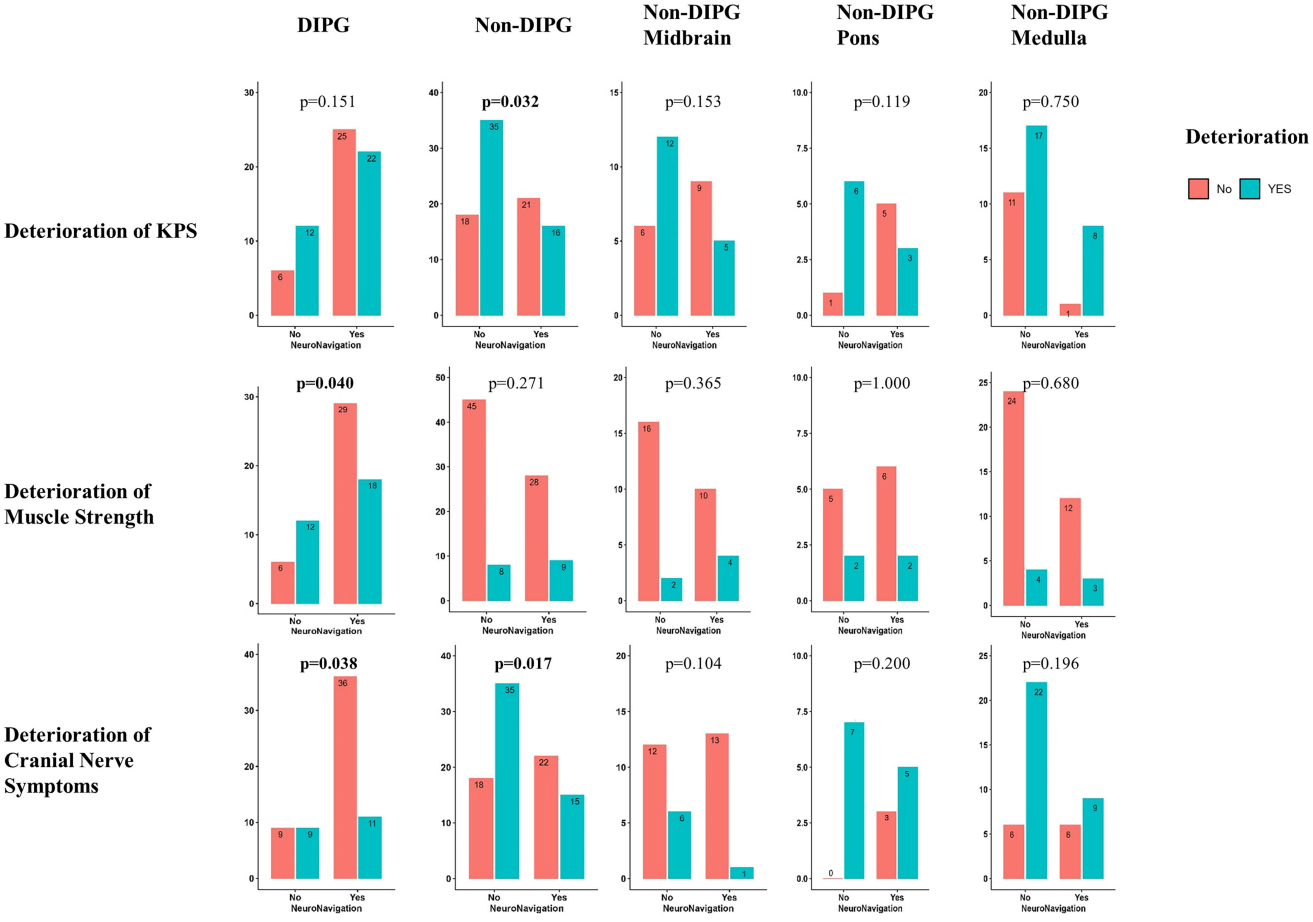
Stratified illustration of differences in deterioration of quality of life between NN group and non-NN group.

It was observed that patients with DIPG had less percentage of deterioration of muscle strength (p=0.040) and cranial nerve function (p=0.038) in the NN group. Moreover, patients with non-DIPG tumors had a lower percentage of deterioration of KPS (p=0.032) and cranial nerve function (p=0.017) in the NN group. The detailed stratified information is illustrated in **Figure 2**.

Considering the effects of age on the outcomes, we compared the outcomes in various groups depending on age, but no significant difference was observed in the subgroups (**Table S1**). Furthermore, logistic regressions were performed to investigate the effects of other factors on the above outcomes (**Table S2-S7**). It was suggested that the usage of NN is an independent protective factor for the deterioration of KPS (p=0.040) and cranial nerve function (p=0.026) in non-DIPG patients and for the deterioration of muscle strength in DIPG patients (p=0.009).

### Higher EOR is an independent factor for better prognosis in DIPG.

As NN could significantly improve the EOR without obvious neurofunction impairment, survival analysis was performed on included patients to determine the long-term effects of NN. Due to the favorable prognosis of non-DIPG patients and this study’s relatively short follow-up duration, only a few cases reached the destination. Therefore, the analysis was only conducted on DIPG patients.

**Figure 3:**
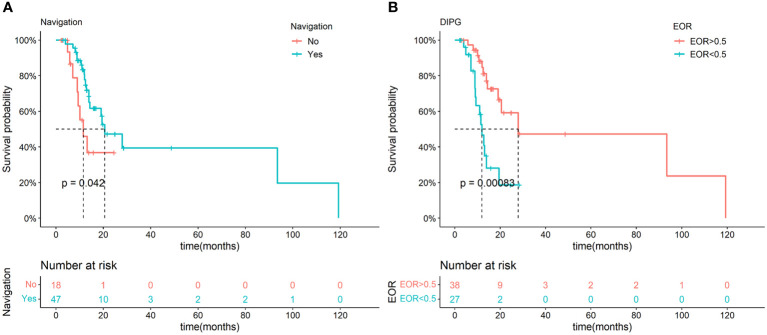
Kaplan-Meier analysis of NN and different EOR in the DIPG groups. Significant difference in OS was observed between NN and non-NN groups in DIPG patients **(A)**. Significant difference in OS was observed between different EOR groups in DIPG patients **(B)**.

According to the Kaplan-Meier analysis, NN showed a positive effect on OS in DIPG (p=0.042, **Figure 3A**), but the effect was not significant in multivariate Cox regression (p=0.294). Interestingly, EOR also showed a positive association with OS in DIPG (p=0.008) in the univariate Cox regression, and the higher EOR was found to be independently related to better prognoses in DIPG patients in the multivariate Cox regression (**Table S8**).

Since the suggested EOR for DIPGs was not confirmed before, we determined the best cutoff values of EOR that can maximally differentiate the survival condition. The best cutoff values of EOR turned out to be 0.50 in DIPG patients by the ROC curve. A significant difference was observed in various EOR groups according to the Kaplan-Meier analysis (p<0.001, **Figure 3B**). Subgroups with an EOR less than and more than 0.5 also showed significant differences in OS, and were independently related to survival in the multivariate Cox regression (P=0.008, **Table S8**). Moreover, the usage of NN is positively correlated with a higher possibility of having an EOR of not less than 0.5 (p<0.001, **Table S9**). Thus, NN may improve the DIPG patient’s prognosis by increasing the EOR.

## Discussion

As NN is a widely used assistive technique with both capacities and short backs, and its value in surgery on BSG is still inadequately investigated, it is of importance to find out whether the assistance of NN can improve surgical outcomes of BSGs (26). In this study, we compared EOR, short-term postoperative quality of life and survival condition of BSG patients who underwent surgical treatment with and without NN. It was found that with the assistance of NN, EOR was higher and fewer patients suffered a decrease in short-term postoperative quality of life. Also, the survival of DIPG patients was found to be better in the NN subgroup. It may be the first study with a comparative approach and relatively large scale to demonstrate NN’s application value in BSG surgery. We hope that these results can provide a reference for further improvement of BSG surgery.

EOR is significantly increased with the assistance of NN, consistent in the DIPG subgroup and non-DIPG subgroup, showing the practical value of intraoperative imaging guidance regardless of surgical strategies (26–28). Neurosurgeons may tend to be conservative in resecting tumors without accurate spatial guidance, presumably because of concerns about protecting neurological function. In turn, knowing the actual location and its relation to surgical boundaries and functional structures gives the neurosurgeons more supportive information and thus confidence in intraoperative decision-making and a consequential higher degree of resection (28, 29).

In this study, we found that with the assistance of NN, while EOR was improved, the incidence of deterioration of short-term life quality after surgery did not increase; on the contrary, part of them was reduced. Deterioration of cranial nerve function was found to be less common in the NN group in non-DIPGs. This trend was also observed in the DIPG group, but the NN was not an independent protective factor. NN could provide more accurate access to the safe entry zone and judgment of tumor boundaries during lesion exposure, which could avoid unnecessary damage and pulling on surrounding structures. Thus, the cranial nerve function was protected well with the assistance of NN in the BSGs surgery. The incidence of deterioration of muscle strength was also observed to decrease with the assistance of NN in DIPG patients. DIPG lesions were reported to have a close relationship with CSTs, and even CSTs pass through the area of lesions in a proportion of cases (14). With the assistance of DTT merged NN, not only CSTs outside the tumor were protected by a clearer indication of tumor boundaries, but also CSTs inside the tumor were protected by NN that can indicate the relationship between the current operational position and their spatial position. We speculated that these might be the reasons for less muscle deterioration in the NN subgroup.

NM is considered the golden criteria for reflecting neurological functions during operation, and NM was reported to be useful in the intraoperative protection of neurological functions (30–34). In this study, NM was used in all operations. However, an abnormal signal detected by NM indicates that events have already taken place, causing damage to critical fiber tracts (23, 31, 32). NN, on the other hand, provides valuable information about the spatial layout of the tracts prior to such events, which could be instrumental in protecting patients’ quality of life. The simultaneous use of NN and NM during brainstem glioma (BSG) surgery would provide information from multiple dimensions regarding resectable boundaries, ultimately resulting in better surgical outcomes.

Considering the above results, NN is valuable in achieving a balance between EOR and the protection of neurological function in BSG surgery. Thus, it is proper to recommend NN as a necessary assistive technique for brainstem glioma surgery.

In addition to NN, several other techniques have also made significant contributions to the development of BSG surgery, such as the use of fluorescein, indocyanine green (ICG), and 5-aminolevulinic acid (5-ALA) which can penetrate the blood-brain barrier and concentrate in the tumor tissue (12, 35). These visualization methods can guide surgeons in determining the tumor boundary in real-time during surgery. However, they have limitations in visualizing vital fiber bundles located within the glioma, which could cause harm to neurological function during surgery. The use of DTT-merged NN could complement these limitations. It is important to acknowledge that each technique has its own advantages and disadvantages. Therefore, integrating multiple techniques during surgery may lead to better outcomes and this is the direction of our future research.

Reports show that higher EOR is related to better prognosis in supratentorial gliomas (26, 36). For non-DIPG, especially low-grade gliomas, higher EOR or complete resection is related to better control of the tumor (37). While EOR’s value in DIPG is inconclusive. DIPG is considered one of the most malignant intracranial tumors where partial resection or cytoreductive surgery was not considered to improve the prognosis of patients significantly and may cause deterioration of neurological dysfunction. Radiotherapy with or without chemotherapy or targeted therapy followed by biopsy was considered a standard treatment for DIPG(6). We observed a positive correlation between EOR and prolonged OS in this study. The value of EOR in DIPG treatment is inadequately reported, which may be a consequence of the prevalence of low EOR and the high risk of surgery-related complications. The findings in this study indicate that pursuing a more proper EOR is considerable under current surgical and assistive techniques contexts. Although our results require further datasets and prospective research to validate, the role of surgical treatment on DIPG undoubtedly needs to be further carefully studied and re-discussed.

During operations, verifications of the NN-guided position of anatomical landmarks were periodically performed. It is observed that spatial shift in the area of the brainstem is insignificant, and we have reported that tractography-merged NN could be comfortably utilized as a reliable tool in surgery for brainstem gliomas (14, 33). We speculate that the difference between this finding and the situation reported in supratentorial glioma surgery is due to several factors (23, 38). First, the brainstem and surrounding tissue edema is usually not severe in BSG patients. Second, hydrocephalus due to obstruction is often relieved by a shunt before surgery. Third, because the brainstem is located in the midline and the surrounding cisterna is symmetrical and broad, removal of CSF during exposure of the lesion will not cause intracranial pressure imbalance. Fourth, pulling surrounding tissues, such as the cerebellum or temporal lobe, during the exposure of lesions is not easy to cause brainstem displacement (39). Thus, NN maintained its accuracy in brainstem surgery.

## Limitations

This study has limitations. First, this study was performed based on retrospective data, which is less convincing than registered prospective studies. Second, there may be selection bias because surgery with NN and the neurosurgeons were not randomized. Third, all cases underwent surgery under NM, so it is impossible to compare the advantages and disadvantages of the two technologies. Fourth, other techniques, such as ICG and 5-ALA, were not included in this study because of the retrospective nature, making it impossible to compare NN with these techniques. Fifth, the relationship between higher EOR and favorable prognosis in DIPG needs to be further investigated due to the uncontrollable confounding factors in surgery.

## Conclusion

In conclusion, with the assistance of NN, BSG surgery achieved higher EOR without deteriorating patients’ symptoms. In addition, higher EOR may benefit DIPG patients with better survival. Thus, NN has significant value in BSG surgery.

## Data availability statement

The original contributions presented in the study are included in the article/**Supplementary Material**. Further inquiries can be directed to the corresponding author.

## Ethics statement

The studies involving human participants were reviewed and approved by Institutional Review Board of Beijing Tiantan Hospital, Capital Medical University. Written informed consent to participate in this study was provided by the participants’ legal guardian/next of kin.

## Author contributions

Conception and design: MZ, XX, LZ. Collection and assembly of data: MZ, XX, GG, PZ, WW, YW, CP, LW, HL. Data analysis and interpretation: MZ, XX. Manuscript writing: MZ, XX. Final approval of manuscript: All authors. Study supervision: All authors. All authors contributed to the article and approved the submitted version.
