# Supplementary material for: Role of neuronavigation in the surgical management of brainstem gliomas
Source: Front Oncol. 2023 May 2;13:1159230. doi: 10.3389/fonc.2023.1159230 (PMC10185888; doi:10.3389/fonc.2023.1159230)
Supplement: Supplementary file 1 [file DataSheet_1.docx]

Supplementary Material

**Role of Neuronavigation in the surgical management of Brainstem gliomas**

Mingxin Zhang, M.D. ^1*^, Xiong Xiao, M.D. ^1^*, Guocan Gu, M.D. ^1^, Peng Zhang, M.D. ^1^, Wenhao Wu, M.D. ^1^, Yu Wang, M.D. ^1^, Changcun Pan, M.D. ^1^, Liang Wang, M.D. ^1^, Huan Li, M.D. ^1^, Zhen Wu, M.D. PhD. ^1^, Junting Zhang, M.D. ^1^, Liwei Zhang, M.D. PhD. ^1,2,3^,

^1^ Department of Neurosurgery, Beijing Tiantan Hospital, Capital Medical University, 100070, Beijing, P.R.China.

^2^ China National Clinical Research Center for Neurological Diseases (NCRC-ND), 100070, Beijing, China.

^3^Beijing Neurosurgical Institute, Beijing Tiantan Hospital, Capital Medical University, 100070, Beijing, China.

* **These authors contributed to this work equally.**

**Corresponding author:** Liwei Zhang. Department of Neurosurgery, Beijing Tiantan Hospital, Capital Medical University.

119, Nansihuan West Road. 100070, Beijing, China

Tel: 86-10-67098431

Fax: 86-10-67051377

**Supplemental Table 1**: Postoperative functional deterioration in different age groups.

|  | Age<14yr | Age>14yr | P |
| --- | --- | --- | --- |
| ***All Patients*** |  |  |  |
| ***KPS*** |  |  | 0.341 |
| Deterioration | 38 (59.4%) | 47 (51.6%) |  |
| No deterioration | 26 (40.6%) | 44 (48.4%) |  |
| ***Muscle strength*** |  |  | **0.047** |
| Deterioration | 25 (39.1%) | 22 (24.2%) |  |
| No deterioration | 39 (60.9%) | 69 (75.8%) |  |
| ***Cranial nerve dysfunction*** |  |  | **0.010** |
| Deterioration | 21 (32.8%) | 49 (53.8%) |  |
| No deterioration | 43 (67.2%) | 42 (46.2%) |  |
| ***DIPG*** |  |  |  |
| ***KPS*** |  |  | 0.429 |
| Deterioration | 24 (55.8%) | 10 (45.5%) |  |
| No deterioration | 19 (44.2%) | 12 (54.5%) |  |
| ***Muscle strength*** |  |  | 0.544 |
| Deterioration | 21 (48.8%) | 9 (40.9%) |  |
| No deterioration | 22 (51.2%) | 13 (59.1%) |  |
| ***Cranial nerve dysfunction*** |  |  | 0.485 |
| Deterioration | 12 (27.9%) | 8 (36.4%) |  |
| No deterioration | 31 (72.1%) | 14 (63.6%) |  |
| ***Non-DIPG*** |  |  |  |
| ***KPS*** |  |  |  |
| Total |  |  | 0.291 |
| Deterioration | 14 (66.7%) | 37 (53.6%) |  |
| No deterioration | 7 (33.3%) | 32 (46.4%) |  |
| Midbrain (N=32) |  |  | 1.000 |
| Deterioration | 4 (50%) | 13 (54.2%) |  |
| No deterioration | 4 (50%) | 11 (45.8%) |  |
| Pons (N=15) |  |  | - |
| Deterioration | 0 | 9 (60%) |  |
| No deterioration | 0 | 6（40%） |  |
| Medulla (N=43) |  |  | 0.178 |
| Deterioration | 10 (76.9%) | 15 (50%) |  |
| No deterioration | 3 (23.1%) | 15 (50%) |  |
| ***Muscle strength*** |  |  |  |
| Total |  |  | 1.000 |
| Deterioration | 4 (19.0%) | 13 (18.8%) |  |
| No deterioration | 17 (81.0%) | 56 (81.2%) |  |
| Midbrain (N=32) |  |  | 0.625 |
| Deterioration | 2 (25.0%) | 4 (16.7%) |  |
| No deterioration | 6 (75.0%) | 20 (83.3%) |  |
| Pons (N=15) |  |  | - |
| Deterioration | 0 | 4 (26.7%) |  |
| No deterioration | 0 | 11（73.3%） |  |
| Medulla (N=43) |  |  | 1.000 |
| Deterioration | 2 (15.4%) | 5 (16.7%) |  |
| No deterioration | 11 (84.6%) | 25 (83.3%) |  |
| ***Cranial nerve dysfunction*** |  |  |  |
| Total |  |  | 0.181 |
| Deterioration | 9 (42.9%) | 41 (59.4%) |  |
| No deterioration | 12 (57.1%) | 28 (40.6%) |  |
| Midbrain (N=32) |  |  | 1.000 |
| Deterioration | 2 (25.0%) | 5 (20.8%) |  |
| No deterioration | 6 (75.0%) | 19 (79.2%) |  |
| Pons (N=15) |  |  | - |
| Deterioration | 0 | 12 (80.0%) |  |
| No deterioration | 0 | 3（20.0%） |  |
| Medulla (N=43) |  |  | 0.079 |
| Deterioration | 7 (53.8%) | 24 (80%) |  |
| No deterioration | 6 (46.2%) | 6 (20.0%) |  |

**Supplemental Table 2**: Univariate and multivariate logistic regression about the deterioration of KPS in DIPG patients.

|  | Univariate |  | Multivariate |  |
| --- | --- | --- | --- | --- |
|  | OR | p | OR | p |
| Age | 0.999 | 0.941 | 0.992 | 0.683 |
| Gender | 0.915 | 0.859 | 0.932 | 0.893 |
| H3K27M mutation | 0.742 | 0.644 |  |  |
| Navigation | 0.440 | 0.156 |  |  |
| Preoperative KPS | 1.044 | 0.024 | 1.045 | 0.023 |
| Preoperative tumor volume | 1.000 | 0.888 |  |  |
| Preoperative cranial nerve dysfunction | 0.348 | 0.148 |  |  |
| Preoperative MS | 1.444 | 0.190 |  |  |
| Excision degree | 0.197 | 0.214 |  |  |

**Supplemental Table 3**: Univariate and multivariate logistic regression about the deterioration of KPS in non-DIPG patients.

|  | Univariate |  | Multivariate |  |
| --- | --- | --- | --- | --- |
|  | OR | p | OR | p |
| Age | 1.001 | 0.943 | 0.994 | 0.661 |
| Gender | 1.504 | 0.341 | 1.398 | 0.474 |
| H3K27M mutation | 1.818 | 0.213 |  |  |
| Navigation | 0.392 | 0.034 | 0.379 | 0.040 |
| Preoperative KPS | 1.063 | 0.008 | 1.064 | 0.008 |
| Location* | 1.250 | 0.615 |  |  |
| Preoperative tumor volume | 1.000 | 0.920 |  |  |
| Preoperative cranial nerve dysfunction | 1.037 | 0.932 |  |  |
| Preoperative MS | 1.617 | 0.167 |  |  |
| Excision degree | 0.186 | 0.156 |  |  |
| Choux classification* | 0.869 | 0.743 |  |  |

*We regard the midbrain and diffuse type as control groups in the Location and Choux classification, respectively.

**Supplemental Table 4**: Univariate and multivariate logistic regression about the deterioration of muscle strength in DIPG patients.

|  | Univariate |  | Multivariate |  |
| --- | --- | --- | --- | --- |
|  | OR | P | OR | P |
| Age | 0.999 | 0.967 | 1.015 | 0.522 |
| Gender | 0.591 | 0.298 | 0.315 | 0.060 |
| H3K27M mutation | 5.600 | 0.036 | 30.538 | 0.004 |
| Preoperative KPS | 1.023 | 0.195 |  |  |
| Navigation | 0.310 | 0.045 | 0.113 | 0.009 |
| Preoperative tumor volume | 1 | 0.199 |  |  |
| Excision degree | 0.039 | 0.022 |  |  |
| Preoperative cranial nerve dysfunction | 1.625 | 0.477 |  |  |
| Preoperative MS | 1.836 | 0.054 |  |  |

**Supplemental Table 5**: Univariate and multivariate logistic regression about the deterioration of muscle strength in non-DIPG patients.

|  | Univariate |  | Multivariate |  |
| --- | --- | --- | --- | --- |
|  | OR | P | OR | P |
| Age | 1.008 | 0.647 |  |  |
| Gender | 1.245 | 0.688 |  |  |
| H3K27M mutation | 1.351 | 0.598 |  |  |
| Preoperative KPS | 0.980 | 0.357 |  |  |
| Navigation | 1.808 | 0.275 |  |  |
| Preoperative tumor volume | 1 | 0.754 |  |  |
| Excision degree | 0.219 | 0.299 |  |  |
| Location* | 1.014 | 0.980 |  |  |
| Preoperative cranial nerve dysfunction | 1.020 | 0.971 |  |  |
| Preoperative MS | 0.681 | 0.310 |  |  |
| Choux classification* | 0.381 | 0.085 |  |  |

*We regard the midbrain and diffuse type as control groups in the Location and Choux classification, respectively.

**Supplemental Table 6**: Univariate and multivariate logistic regression about the deterioration of cranial nerve symptoms in DIPG patients.

|  | Univariate |  | Multivariate |  |
| --- | --- | --- | --- | --- |
|  | OR | P | OR | P |
| Age | 1.032 | 0.106 | 1.027 | 0.181 |
| Gender | 0.893 | 0.835 | 0.947 | 0.924 |
| H3K27M mutation | 0.553 | 0.369 |  |  |
| Preoperative KPS | 1.012 | 0.525 |  |  |
| Navigation | 0.306 | 0.042 | 0.339 | 0.069 |
| Preoperative tumor volume | 1 | 0.871 |  |  |
| Excision degree | 6.720 | 0.178 |  |  |
| Preoperative cranial nerve dysfunction | 0.737 | 0.660 |  |  |
| Preoperative MS | 1.356 | 0.337 |  |  |

**Supplemental Table 7**: Univariate and multivariate logistic regression about the deterioration of cranial nerve symptoms in non-DIPG patients.

|  | Univariate |  | Multivariate |  |
| --- | --- | --- | --- | --- |
|  | OR | P | OR | P |
| Age | 1.026 | 0.063 | 1.028 | 0.102 |
| Gender | 1.152 | 0.740 | 1.569 | 0.418 |
| H3K27M mutation | 1.241 | 0.644 |  |  |
| Preoperative KPS | 1.015 | 0.409 |  |  |
| Navigation | 0.351 | 0.018 | 0.299 | 0.026 |
| Preoperative tumor volume | 1 | 0.097 |  |  |
| Excision degree | 1.284 | 0.828 |  |  |
| Location* | 10.238 | <0.001 | 14.651 | <0.001 |
| Preoperative cranial nerve dysfunction | 0.942 | 0.887 |  |  |
| Preoperative MS | 2.008 | 0.060 |  |  |
| Choux classification* | 1.152 | 0.740 |  |  |

*We regard the midbrain and diffuse type as control groups in the Location and Choux classification, respectively.

**Supplemental Table 8**: Univariate and multivariate COX regression about the OS for DIPG patients.

|  | Univariate |  | Multivariate |  |
| --- | --- | --- | --- | --- |
|  | HR | P | HR | P |
| Age | 0.982 | 0.231 | 0.978 | 0.141 |
| Gender | 1.651 | 0.222 | 1.792 | 0.167 |
| H3K27M mutation | 2.514 | 0.136 |  |  |
| Preoperative KPS | 0.988 | 0.308 |  |  |
| Postoperative KPS | 0.989 | 0.276 |  |  |
| Navigation | 0.423 | 0.048 | 0.605 | 0.294 |
| Preoperative tumor volume | 1 | 0.565 |  |  |
| Postoperative tumor volume | 1 | 0.068 |  |  |
| Excision degree* | 0.059 | 0.008 |  |  |
| Excision degree>0.50* | 0.282 | 0.002 | 0.315 | 0.008 |
| Hydrocephalus | 1.560 | 0.417 |  |  |
| Preoperative cranial nerve dysfunction | 5.664 | 0.090 |  |  |
| Preoperative MS | 0.988 | 0.954 |  |  |
| Postoperative MS | 0.892 | 0.421 |  |  |
| Deterioration of MS | 1.439 | 0.355 |  |  |
| Deterioration of cranial nerve symptom | 0.679 | 0.388 |  |  |
| Deterioration of KPS | 1.113 | 0.787 |  |  |

*50% was defined as the cutoff value of the excision degree for DIPG patients. The p and HR value of excision degree in the multivariate COX regression is 0.027 and 0.08, respectively.

**Supplemental Table 9**: The usage of NN is correlated with a higher EOR subgroup.

|  | Navigation | Non-navigation | P value |
| --- | --- | --- | --- |
| ***DIPG*** |  |  | <0.001 |
| EOR>0.50 | 34 (72.3%) | 4 (22.2%) |  |
| EOR<0.50 | 13 (27.7%) | 14 (77.3%) |  |
| ***Non-DIPG*** |  |  | 0.007 |
| EOR>0.50 | 34 (91.8%) | 36 (67.9%) |  |
| EOR<0.50 | 3 (8.2%) | 17 (32.1%) |  |


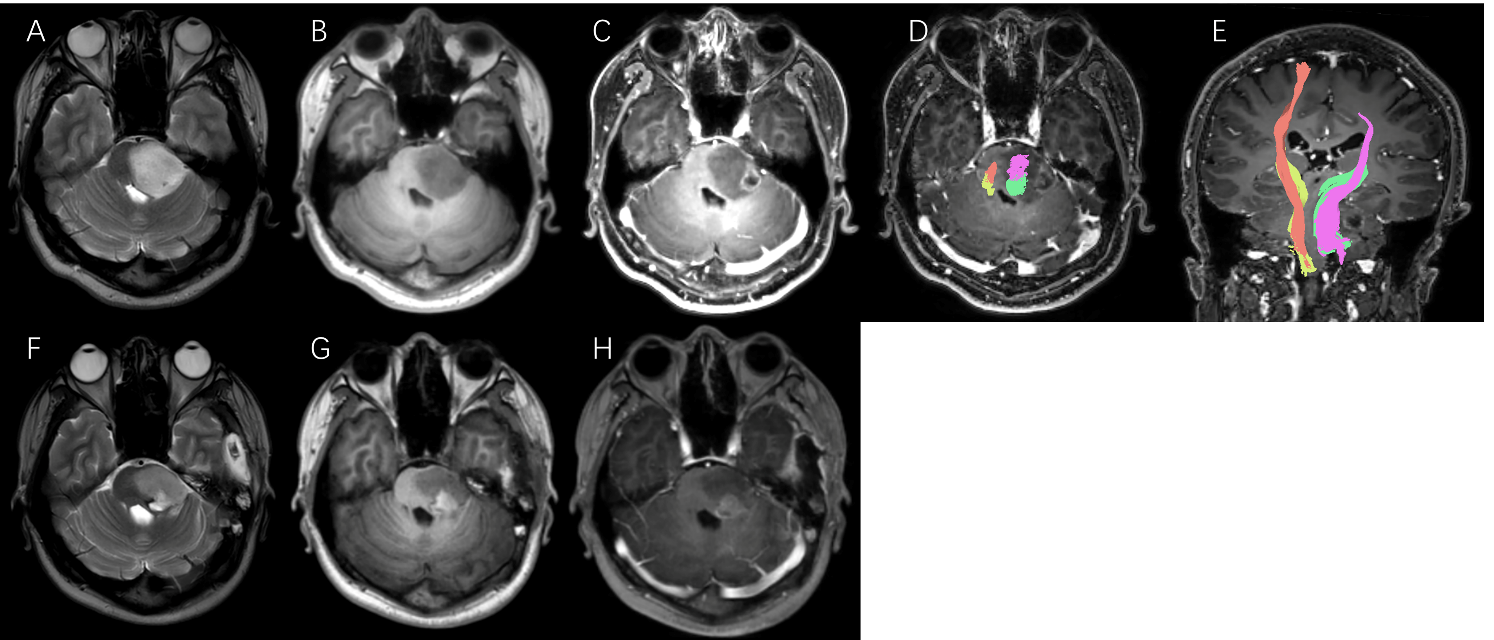


Figure S1 This patient, a 20-year-old man, was admitted to the hospital with numbness of the right limb for one month. He received cytoreductive surgery with the assistance of neuronavigation and intraoperative electroneurophysiological monitoring in 2022-03-21, and followed by radiotherapy and chemotherapy. Picture shows a characteristic radiological features of diffuse brainstem glioma with an obvious enhanced area and part of the vital fiber within the tumor (A-E). With the guidance of neuronavigation and preoperative DTI, retrosigmoid approach was selected and the enhanced lesion was resected selectively without the damage of motor function (F-H). This patient was in stable neurofunctional status 9 months after surgery.


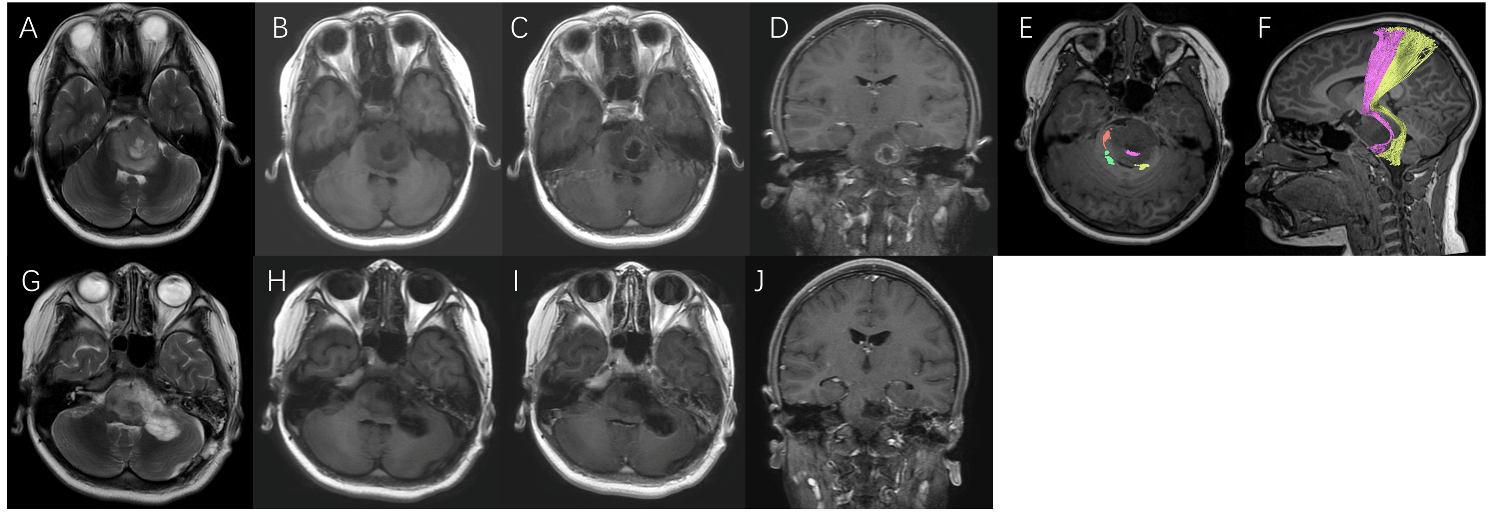


Figure S2 This patient, a 9-year-old boy, was admitted to the hospital with confusion and hemiplegia of the right limb for one month. He received cytoreductive surgery with the assistance of neuronavigation and intraoperative electroneurophysiological monitoring in 2022-06-01, and followed by radiotherapy and immunotherapy. Picture shows a characteristic radiological feature of DIPG with an obvious enhanced area and the vital fiber tracts were pushed aside by the tumor, which makes cytoreductive surgery possible and safe (A-F). With the guidance of neuronavigation and preoperative DTI, retrosigmoid approach was selected and the ring-enhancement lesion was resected without obvious damage of critical fibers. Patient showed a relief of presurgical symptoms after surgery (G-J). Furthermore, 4 months after surgery, although this patient was unable to walk totally unassitsted, his neurofunction was recovered than preoperative status.
